# Use of a bioinformatic-assisted primer design strategy to establish a new nested PCR-based method for *Cryptosporidium*

**DOI:** 10.1186/s13071-017-2462-4

**Published:** 2017-10-23

**Authors:** Anson V. Koehler, Pasi K. Korhonen, Ross S. Hall, Neil D. Young, Tao Wang, Shane R. Haydon, Robin B. Gasser

**Affiliations:** 1 Department of Veterinary Biosciences, Melbourne Veterinary School, Faculty of Veterinary and Agricultural Sciences, The University of Melbourne, Parkville, VIC 3010 Australia; 2 Melbourne Water, Docklands, VIC 3001 Australia

**Keywords:** *Cryptosporidium*, Bioinformatics, Large subunit of nuclear ribosomal RNA gene (*LSU*), D8 domain, Primers, Polymerase chain reaction (PCR)

## Abstract

**Background:**

The accurate tracking of *Cryptosporidium* in faecal, water and/or soil samples in water catchment areas is central to developing strategies to manage the potential risk of cryptosporidiosis transmission to humans. Various PCR assays are used for this purpose*.* Although some assays achieve specific amplification from *Cryptosporidium* DNA in animal faecal samples, some do not. Indeed, we have observed non-specificity of some oligonucleotide primers in the small subunit of nuclear ribosomal RNA gene (*SSU*), which has presented an obstacle to the identification and classification of *Cryptosporidium* species and genotypes (taxa) from faecal samples.

**Results:**

Using a novel bioinformatic approach, we explored all available *Cryptosporidium* genome sequences for new and diagnostically-informative, multi-copy regions to specifically design oligonucleotide primers in the large subunit of nuclear ribosomal RNA gene (*LSU*) as a basis for an effective nested PCR-based sequencing method for the identification and/or classification of *Cryptosporidium* taxa.

**Conclusion:**

This newly established PCR, which has high analytical specificity and sensitivity, is now in routine use in our laboratory, together with other assays developed by various colleagues. Although the present bioinformatic workflow used here was for the specific design of primers in nuclear DNA of *Cryptosporidium*, this approach should be broadly applicable to many other microorganisms.

**Electronic supplementary material:**

The online version of this article (10.1186/s13071-017-2462-4) contains supplementary material, which is available to authorized users.

## Background

Animal faecal contamination in drinking water catchment areas is a global concern, and has the potential to lead to waterborne outbreaks of human gastrointestinal diseases including cryptosporidiosis, caused by members of the genus *Cryptosporidium* [1–5]. The identification and classification of *Cryptosporidium* species and genotypes (taxa) in faecal, water and/or soil samples in water catchment areas is central to assessing the risk of zoonotic transmission of cryptosporidiosis and to developing strategies to monitor and manage this risk [1–5].

As the oocysts and other developmental stages of different *Cryptosporidium* taxa cannot be unequivocally identified or differentiated microscopically, molecular tools can be used for their specific and genotypic identification and classification [6, 7]. A widely-used approach has been nested PCR-based sequencing or restriction fragment length polymorphism (RFLP) of particular gene markers within the *actin*, *cowp*, *hsp*70 and/or small subunit of nuclear ribosomal RNA (*SSU*) genes (e.g. [3, 7–9]). The sequencing of *SSU* has been particularly commonly used, because of its multi-copy nature [10], making PCR analytically sensitive, and because it contains diagnostically informative markers for the classification of *Cryptosporidium* taxa (cf. [3, 9, 11–13]). Indeed, to date, more than 34 species and 40 genotypes of *Cryptosporidium* have been classified using partial *SSU* sequence data [3, 5, 14–17].

Various PCR protocols are used for the amplification of regions within *SSU* (e.g. [8, 11, 12, 18])*.* When applied to faecal DNA samples from humans, most of these protocols appear to achieve relatively specific amplification from *Cryptosporidium* DNA for direct amplicon-sequencing. However, although not necessarily alluded to in the published literature, some PCR assays do not achieve high specificity when applied to faecal DNA samples originating from various animals, indicating that some primer sets designed are not entirely specific for *Cryptosporidium*. Indeed, we have observed ‘cross-amplification’ of *SSU* from DNA of some *Cryptosporidium*-related apicomplexans (e.g. [19]) as well as selected alveolates and/or dinoflagellates in animal faeces employing primers originally designed to *Cryptosporidium* spp. (Koehler et al., unpublished findings). Although we have designed and tested numerous new primer pairs, we did not achieve the desired level of specificity. Other primer sets to *SSU* [18] were designed to improve the sensitivity of primers over the leading primer set [8] by decreasing the regions targeted in the primary and secondary (nested) PCR amplification steps. Here, we used a novel bioinformatic approach to explore all available *Cryptosporidium* genome sequences (cf. [20]) for new and diagnostically-informative, multi-copy regions that would allow us to specifically design oligonucleotide primers to establish a nested PCR-based sequencing method with high analytical specificity and sensitivity for the identification and/or classification of *Cryptosporidium* species and genotypes.

## Methods

### Genomic DNAs from faecal deposits, and grouping of samples based on results from PCR-based sequencing of *SSU*

In the present study, we used a total of 332 genomic DNA samples from faecal samples from humans and 13 different animal species (mammals and birds) available from previous studies [2, 3, 21–23] or provided by colleagues (cf. Additional file 1: Table S1). The host origin of each sample was known or inferred using an authoritative field guide [24], and verified, as required, by PCR-based sequencing of a region of the mitochondrial cytochrome *b* gene from faecal DNA (cf. [25]). Genomic DNA was usually extracted directly from 0.25 g of each faecal sample using the PowerSoil kit (MoBio, Carlsbad, CA, USA) as described previously [3]; published evidence demonstrates that this method removes PCR-inhibitory components from faecal samples [26].

Genomic DNAs were subjected to PCR-based sequencing of *SSU* as described previously [21]. In brief, primary PCR (~850 bp) was conducted using primers XF2 (forward: 5′-GGA AGG GTT GTA TTT ATT AGA TAA AG-3′) and XR2 (reverse: 5′-AAG GAG TAA GGA ACA ACC TCC A-3′) (cf. [8]), followed by a secondary (nested) PCR (215 bp) using primers pSSUf (forward: 5′-AAA GCT CGT AGT TGG ATT TCT GTT-3′) and pSSUr (reverse: 5′-ACC TCT GAC TGT TAA ATA CRA ATG C-3′). All oligonucleotide primers (degenerate nucleotide positions indicated with an International Union of Pure and Applied Chemistry (IUPAC) code) were synthesized by GeneWorks, Thebarton, Australia.

**Fig. 1:**
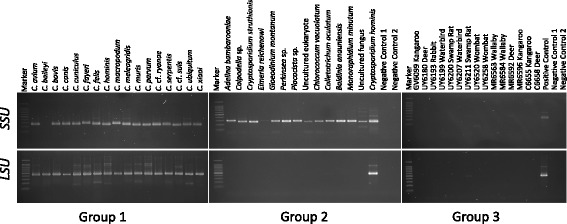
Agarose gel showing results for each of the three groups of (individual) genomic DNA samples produced by nested PCR of regions of the small (*SSU*) and large (*LSU*) subunits of the nuclear ribosomal RNA genes. *Group 1*: Well-defined ‘positive’ samples containing DNA of particular *Cryptosporidium* taxa, with no ‘cross-amplification’. *Group 2*: Representative samples with substantial cross-amplification but no evidence of the presence of *Cryptosporidium*. *Group 3*: Representative ‘negative’ samples with no cross-amplification or evidence of the presence of *Cryptosporidium* DNA (cf. Methods section). The marker is a 100 bp ladder, plus a 1500 bp band (top). No-template controls were included in primary and secondary PCR runs (negative controls 1 and 2, respectively). For full description of samples and methods, see Methods section

For primary PCR, the cycling protocol was: 94 °C for 5 min (initial denaturation), followed by 30 cycles of 94 °C for 45 s (denaturation), 45 °C for 2 min (annealing) and 72 °C for 1.5 min (extension), with a final extension of 72 °C for 10 min. For secondary PCR, the protocol was: 94 °C for 5 min, followed by 35 cycles of 94 °C for 30 s, 55 °C for 30 s and 72 °C for 30 s, with a final extension of 72 °C for 10 min. Amplicons were individually treated with ExoSAP-IT (Affymetrix, Santa Clara, CA, USA), according to the manufacturer’s instructions, and then subjected to bi-directional automated sequencing (BigDye® Terminator v.3.1 chemistry, Applied Biosystems, Foster City, CA, USA) using the same primers employed in the secondary (nested) PCR. Sequence quality was verified by comparison with corresponding electropherograms using the program Geneious v.9.1.4 [27]. Each *SSU-*derived sequence obtained was compared by BLASTn analysis with publicly available data (GenBank) (cf. [2, 3]). Based on the results, five distinct groups of genomic DNA samples were formed (Additional file 1: Table S1):

*Group 1* (well-defined ‘positive’ samples containing DNA of particular *Cryptosporidium* taxa but without ‘cross-amplification’): Samples (*n* = 20; Additional file 1: Table S1; Fig. 1) containing one of the following species: *C. avium*, *C. baileyi*, *C. bovis*, *C. canis*, *C. cuniculus*, *C. fayeri*, *C. felis*, *C. hominis*, *C. macropodum*, *C. meleagridis*, *C. muris*, *C. parvum*, *Cryptosporidium* cf. *ryanae*, *C. serpentis*, *Cryptosporidium* cf. *suis*, *C. ubiquitum*, *C. xiaoi*, *Cryptosporidium* sp. “deer genotype”, *Cryptosporidium* sp. “duck-like genotype”, *Cryptosporidium* cf. *fayeri* EGK1, *Cryptosporidium* sp. “kangaroo genotype I”. *Cryptosporidium* sp. “mink genotype” or *Cryptosporidium* sp. “novel human genotype”.

*Group 2* (samples with substantial cross-amplification and no evidence of the presence of *Cryptosporidium*): Samples (*n* = 13; Additional file 1: Table S1) from which one of the following microorganisms was amplified by PCR of *SSU* and sequenced: *Adelina bambarooniae*, *Colpodella* sp., *Cryptosporidium struthionis* (not recognized as a valid member of the genus *Cryptosporidium* [18]), *Eimeria reichenowi*, *Gloeodinium montanum*, *Perkinsea* sp*.*, *Placocista* sp., “Uncultured eukaryote”, Chlorophytes; *Chlorococcum vacuolatum*, *Colletotrichum aculatum*, *Monoraphidium minutum*, dinoflagellate; *Baldinia anauniensis*; or fungus, “Uncultured fungus” (Fig. 1)*.* These microorganisms have been commonly detected by *SSU*-PCR in a longitudinal study of waterborne pathogens in faecal samples from Australian wildlife (cf. [2, 3]).

*Group 3* (‘negative’ samples without cross-amplification with no evidence of the presence of *Cryptosporidium* DNA)*:* Samples (*n* = 15; Additional file 1: Table S1; Fig. 1) from which no *SSU* amplicons were produced, and for which there was no evidence of PCR inhibition or background amplification in *SSU*-PCR.

*Group 4* (samples with substantial cross-amplification and containing *Cryptosporidium* DNA)*:* Samples (*n* = 9; Additional file 1: Table S1) from human faecal samples known to contain *Cryptosporidium* taxa. Attempts at sequencing the *SSU* locus from these “challenging” samples yielded indeterminate sequence data (not shown). To obtain unambiguous sequence data to classify the *Cryptosporidium* taxa within these samples, we amplified part of the 60 kDa-glycoprotein gene (*gp60*) using a standard method and sequenced the products (cf. [21]).

*Group 5* (samples without cross-amplification and containing DNA of closely-related genotypes or subtypes of *Cryptosporidium*)*:* Samples (*n* = 24; Additional file 1: Table S1) each containing a particular genotype of *C. fayeri*, *C. macropodum*, *C. ryanae* and *C. ubiquitum* (*n* = 15) or a particular subtype of *C. parvum* or *C. hominis* (*n* = 9)*.*

### Bioinformatic workflow for the specific design of new primers

Focused on designing highly specific oligonucleotide primers for use in PCR, we interrogated all publicly available genomic data available for *Cryptosporidium* species for multi-copy DNA (copy number = 4; [10]), and identified *LSU* as the key candidate with the greatest potential to achieve high analytical sensitivity. As only one full-length *LSU* sequence (accession number AF040725; [28]) was available for *C. parvum* in GenBank, we used a bioinformatic approach to identify homologous sequences in other *Cryptosporidium* data sets as well as similar *LSU* sequences in other microorganisms, and created an alignment from which oligonucleotide primers could be designed (described below).

In brief, genome sequence files (FASTA) and the general feature format (GFF) files for available *Cryptosporidium* species (February 2016) as well as relatively ‘closely related’ alveolates and dinoflagellates (for which *LSU* data were available; cf. Additional file 1: Table S1) were downloaded from GenBank, the *Cryptosporidium* Genomic Resources Database release 27 (cryptoDB.org) and the Silva rRNA Database Project (www.arb-silva.de), respectively: i.e. *Cryptosporidium baileyi* (JIBL01000085); *C. hominis* (JRXJ01000009); *C. meleagridis* (JIBK01000002); *C. muris* (DS989727); *C. parvum* (AF040725); *Cryptosporidium* sp. “chipmunk genotype” (JXRN01000036); *Eimeria tenella* (CBUW010002483); *Colpodella angusta* (KU159286); *Perkinsus chesapeaki* (AY305326); *P. olseni* (AF509333); “Uncultured marine dinoflagellate” (FJ032679); *Vitrella brassicaformis* (HM245049); and *Voromonas pontica* (EF681910).

For species for which only genome sequence files were available, modelled repeats were combined with a collection of known repeats in Repbase v.17.02 [29] and soft-masked using RepeatMasker v.open-3.3.0 [30]. Repeats identified as representing *LSU* were selected and reverse-complemented (as required) and then converted into the FASTA format. An alignment of *LSU* sequences (over ~3800 bp) (Additional file 2: Figure S1) was made using MAFFT in Mesquite [31] and transferred to the program Geneious v9.1.4 [27] where outer and inner (nested) pairs of primers were designed to the flanking regions of the variable D8 domain of *LSU* (cf. [32]) with the aid of the program Primer3 (http://primer3.ut.ee/) [33] (Additional file 2: Figure S1). The primers designed were: LSU2040F (forward: 5′-CGA ATA GCG TTA TCT TTG CTA TTT-3′) and LSU3020R (reverse: 5′-GTC TTC CGC GAA GAT CAG-3′), followed by secondary (nested) PCR (~500 bp) using primers LSU2065F (forward: 5′-TTA CCA TGG AAT (C/T)AG TTC AGC-3′) and LSU2557R (reverse: 5′-AAC ACC ATT TTC TGG CCA TC-3′).

### Protocol for the newly established nested PCR

The final method is described in the following: The nested PCR was carried out in 50 μl using a standard reaction buffer, 3.0 mM of MgCl_2,_ 200 μM of each dNTP, 50 pmol of each primer and 1 U of *Taq* polymerase (MangoTaq, Bioline, London, UK). Primary PCR of the D8 region (~1000 bp) of the *LSU* gene was conducted using the primer pair LSU2040F/LSU3020R, followed by secondary (nested) PCR (~500 bp) using the primer pair LSU2065F/LSU2557R. The conditions of the primary PCR were: 94 °C for 5 min (initial denaturation), followed by 35 cycles of 94 °C for 30 s (denaturation), 58 °C for 30 s (annealing) and 72 °C for 50 s (extension), with a final extension of 72 °C for 5 min. The conditions of secondary PCR were the same, except that the extension step was 30 s instead of 50 s. Except for the no-template controls, 2 μl of genomic DNA were added to the primary PCR, from which 1 μl was carried over to the secondary PCR. No-template (negative) controls were included at all steps, and no-template controls were carried over from the primary to the secondary (nested) PCR. A well-known positive control sample (*C. parvum* DNA) was included in each PCR run. The sequencing of *LSU* amplicons was performed (as described for *SSU* amplicons) using primers LSU2065F and LSU2557R.

### Phylogenetic analysis of *LSU* and *SSU* sequence data

Sequences were aligned using the program MAFFT [34], and alignments were manually adjusted using the program Mesquite v.3.10 [31] (cf. Additional file 2: Figure S2). Sequences were then compared with those available in GenBank using BLASTn (NCBI) (see Additional file 1: Tables S2 and S3 for additional sequence data used for comparative purposes). *Colpodella angusta* was chosen as a suitable outgroup due to its proposed close relatedness to *Cryptosporidium* (cf. [19]). Specifically, phylogenetic analysis of sequence data was conducted by Bayesian inference (BI) using Monte Carlo Markov Chain (MCMC) analysis in MrBayes v.3.2.6 [35]. The likelihood parameters set for BI analysis of *LSU* data were based on the Akaike Information Criteria (AIC) test in jModeltest v.2.1.10 [36]. For trees constructed using (partial) *LSU* or *SSU* sequence data, the number of substitutions (Nst) was set at 6, with a gamma-distribution. For *SSU*, the proportion of invariable sites was also considered. Posterior probability (pp) values were calculated by running 2,000,000 generations with four simultaneous tree-building chains. Trees were saved every 100th generation. At the end of each run, the standard deviation of split frequencies was <0.01, and the potential scale reduction factor approached one. A 50% majority rule consensus tree for each analysis was constructed based on the final 75% of trees generated by BI. Analyses were run three times to ensure convergence and insensitivity to priors.

## Results and discussion

### Establishing the new assay using primers designed employing a bioinformatic approach

Using the present bioinformatic workflow (see Methods section), we identified specific regions in *LSU* (flanking the D8 domain) and designed primers LSU2040F (forward: 5′-CGA ATA GCG TTA TCT TTG CTA TTT-3′) and LSU3020R (reverse: 5′-GTC TTC CGC GAA GAT CAG-3′) to amplify a region of ~800 bp in primary PCR, and primers LSU2065F (forward: 5′-TTA CCA TGG AAT YAG TTC AGC-3′) and LSU2557R (reverse: 5′-AAC ACC ATT TTC TGG CCA TC-3′) to amplify a region of ~500 bp in secondary (nested) PCR. Having designed these primer pairs, we proceeded to establish the nested PCR assay, following authoritative guidelines and recommendations for optimization [37]. The final protocol/method is described in the Methods section.

### Performance of the newly established nested PCR

To evaluate the specificity of the PCR assay, we produced *LSU* amplicons from all genomic DNA samples in *Groups 1*–*4* and sequenced them. The PCR and sequencing results for individual samples are given in Additional file 1: Table S1. The sequencing of the amplicons derived from *Group 1*-samples (Fig. 1) yielded sequences that exclusively/specifically represented *Cryptosporidium*. Using the nested *LSU*-PCR, no products of ~500 bp were amplified from any of the samples in *Groups 2* or *3*, showing the specificity of the newly designed primers (Fig. 1). The sequencing of *LSU* amplicons derived from all individual *Group 4*-samples allowed the unequivocal identification of *Cryptosporidium* in each sample, without any complication of background- or cross-amplification, in contrast to results obtained using PCR-based sequencing of *SSU* (Additional file 1: Table S1). In conclusion, *LSU* amplicons produced from individual genomic DNAs were consistently abundant and of the expected size (~500 bp) on agarose gels, and sequences derived from these amplicons consistently and unambiguously matched those of *Cryptosporidium*, which represented a substantial improvement over results achieved previously using *SSU*-PCR (see Methods section). Thus, using the *LSU*-PCR, there was no instance of background- or cross-amplification of a ~500 bp product from any microbial taxon other than *Cryptosporidium* (based on direct sequencing of amplicons). Using serial titration experiments, the analytical sensitivity of the *LSU*-PCR was estimated at < 1 pg of *Cryptosporidium* DNA (data not shown). All results obtained were reproducible (at least three times) on different days.

### Ability of PCR-based sequencing of *LSU* to assign species and/or genotypes of *Cryptosporidium*

Here, we used 24 samples from *Group 5* to assess the ability of PCR-based sequencing of *LSU* to differentiate closely related genotypes or subtypes of select *Cryptosporidium* (cf. Additional file 1: Table S1). We compared the magnitude of sequence variation in *LSU* within each of four species with that in *SSU* for the same species. Specifically, maximum sequence variation in *LSU* within *C. fayeri* (2.6%), *C. macropodum* (0%), *C. ryanae* (5.9%) and *C. ubiquitum* (0.8%), using 2–5 samples per species (cf. Additional file 1: Tables S2 and S3), was for the most part, less than that recorded in *SSU* for the similar samples (5.5%, 1.9%, 3.4% and 3.2%, respectively) (cf. Additional file 2: Figure S2). Subsequently, we used the remaining 9 genomic DNAs from human faecal samples from *Group 5* to assess sequence variability in *LSU* among selected subtypes of both *C. parvum* and *C. hominis*, and then compared the magnitude of variation in *LSU* with that derived from a comparative set of *SSU* sequences representing the same taxa (i.e. species and subtypes) from GenBank. Sequence variation in *LSU* within *C. parvum* (0%) and *C. hominis* (1.0%) was similar to that recorded for the comparative set of *SSU* sequences (0% and 1.8%, respectively) (cf. Additional file 2: Figure S2).

**Fig. 2:**
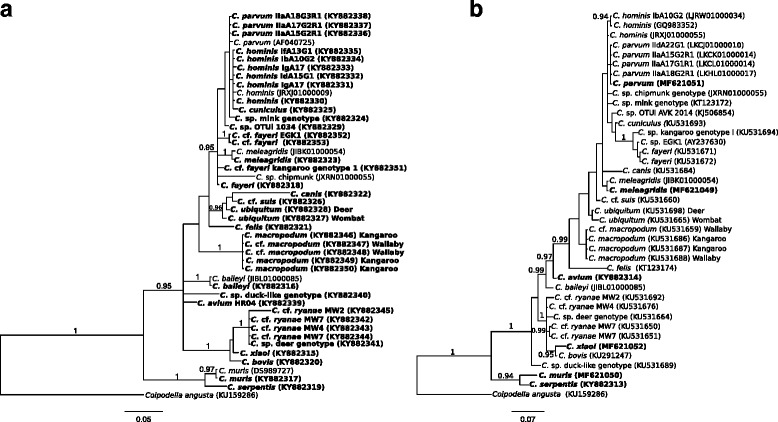
**a** Relationships among *Cryptosporidium* taxa inferred from the phylogenetic analysis of sequence data for the variable D8 domain of the large subunit of nuclear ribosomal RNA gene (*LSU*) by Bayesian inference (BI). **b** Relationships among *Cryptosporidium* taxa inferred from the phylogenetic analysis of sequences from the small subunit of the nuclear ribosomal RNA gene (*SSU*) by Bayesian inference (BI). Posterior probabilities are indicated at all major nodes. Bold-type indicates *Cryptosporidium* species or genotypes characterized from faecal DNA samples in this study. Individual identification codes and accession numbers follow the species or genotype designation. Scale-bar represents the number of substitutions per site. The alveolate *Colpodella angusta* was used as an outgroup

Then, a phylogenetic tree built using the *LSU* sequence data (Additional file 1: Table S4) showed that it could differentiate *Cryptosporidium* variants (genotypes) with similar resolution to the tree constructed using *SSU* data (Additional file 1: Table S5; Figure 2) - the latter locus being that most extensively used for phylogenetic studies of *Cryptosporidium* to date [7, 9]. Subsequently, we assessed whether phylogenetic resolution would differ significantly when *LSU* are used instead of *SSU* data*.* For the important human-affiliated species, *C. hominis* and *C. parvum*, an analysis of the *LSU* data did not provide more resolution than the *SSU* data (also for samples previously characterized using the *gp60* locus; cf. [21]). Although statistically unsupported, genotypes of *C. parvum* and *C. hominis* clustered separately, showing that they can be readily distinguished, both in an alignment of *LSU* sequence data (Additional file 2: Figure S2) and in the *LSU* tree (Fig. 2). Nonetheless, the sequence of the D8 domain of *LSU* does not discern some of the genotypes or subtypes that could be identified/differentiated using the *SSU* locus (cf. Fig. 2). For example, *C. ryanae* was not divided in the *LSU* tree, whereas for *SSU*, multiple *C. ryanae*-like genotypes (e.g. MW1, MW4 and MW7; [2]) could be distinguished. Additionally, differences between genotypes of some *Cryptosporidium* species, such as *C. ubiquitum,* which have several nucleotide alterations in *SSU* in *Cryptosporidium* from wombats compared with more common subtypes [38], were retained when the *LSU* locus was used (see *C. ubiquitum* in Fig. 2). In our opinion, less variation (resolution) might actually be advantageous in some instances, particularly when genetically discriminating among *Cryptosporidium* species, as the D8 domain of *LSU* tends to ‘combine’ closely related genotypes, rather than divide them up. As noted by Xiao et al. [6], it is challenging to predict what degree of genetic variability is needed for the differentiation of species, and the question arises at what point should genotypes and/or subtypes be coined (cf. [38]).

## Conclusions

Here, we have shown the benefit of using a bioinformatics-guided approach for the specific design of primers for PCR. In this case, our focus was on finding regions in multi-copy ribosomal DNA to ensure that any PCR developed would achieve high analytical sensitivity. The laboratory-based evaluations conducted using well-defined groups of genomic (faecal) DNAs showed unequivocally that both analytical specificity and sensitivity of this nested PCR are very high, allowing the genetic characterization and classification of *Cryptosporidium* species and genotypes by phylogenetic means. This newly established PCR is now in routine use in our laboratory, together with other assays originally developed by various colleagues [8, 11]; having tested > 900 animal faecal DNA samples to date, the performance of our nested *LSU*-PCR is entirely consistent with that achieved here and results are reproducible (Wang and Koehler, unpublished findings). The specificity and sensitivity of this new assay need to be continuously monitored over time. It will also be relevant to reassess these parameters if the present assay were deployed to other geographical regions or applied to faecal samples from animal species distinct from those tested herein. Although we have not yet had the opportunity to test genomic DNA from the recently described piscine species of *Cryptosporidium* (e.g. *C. huwi*; [14]), we anticipate that the present *LSU*-PCR assay will specifically amplify DNA from this taxon. We hope that this assay could eventually be added to the currently recommended tool-kit (using markers in the *cowp* and *SSU* genes) for the genetic characterization of *Cryptosporidium* species and genotypes (cf. [7, 39]). In conclusion, although the present bioinformatic workflow was used for the specific design of reagents (primer pairs) nuclear DNA of *Cryptosporidium*, we believe that this approach will be applicable to a wide range of genes in the genomes of many other microorganisms.

## Additional files

Additional file 1: Table S1. Relevant information pertaining to small subunit ribosomal RNA gene (*SSU*) sequences obtained from amplicons produced from selected faecal DNA samples using a nested PCR method ([2]; see Methods section). These DNA samples were employed to assess the analytical specificity of large subunit ribosomal RNA gene (*LSU*) nested PCR assay established in this study (see also Methods section) (cf. Fig. 1). Sequence similarities (90–100%) were calculated with reference to the closest matched sequence in the GenBank database using BLASTn. **Table S2.** Pairwise comparison of sequence difference (%) in the variable D8 domain of the large subunit of the nuclear ribosomal RNA gene (*LSU*) used for the construction of the phylogenetic tree. **Table S3.** Pairwise comparison of sequence difference (%) in the region of the small subunit of the nuclear ribosomal RNA gene (*SSU*) used for the construction of the phylogenetic tree. **Table S4.** Salient information pertaining to the sequences of the variable D8 domain of the nuclear large subunit ribosomal RNA gene (*LSU*) used for the construction of the phylogenetic tree (cf. Fig. 2a). Sequences produced in this study are shown in bold-type. **Table S5.** Salient information pertaining to the sequences from the small subunit of the nuclear ribosomal RNA gene (*SSU*) used for the construction of the phylogenetic tree (cf. Fig. 2b). Sequences produced in this study are shown in bold-type. (XLSX 78 kb)

Additional file 2: Figure S1. Alignment of sequences of the variable D8 domain of the large subunit of nuclear ribosomal RNA gene (*LSU*) representing *Cryptosporidium* and closely related apicomplexans, alveolates and dinoflagellates. Oligonucleotide primers (LSU2040F, LSU3020R; LSU2065F and LSU2557R) designed specifically to regions flanking the variable D8 domain are indicated in green. Nucleotide differences from the majority consensus of the alignment are highlighted. **Figure S2.** Alignment of sequences of the variable D8 domain of the large subunit of nuclear ribosomal RNA gene (*LSU*) representing *Cryptosporidium* derived from 45 faecal DNA samples. Nucleotide differences from the majority consensus of the alignment are highlighted. *Colpodella angusta* was included as an outgroup. (PDF 222 kb)

## Abbreviations

AIC: Akaike information criteria
BI: Bayesian inference
GFF: general feature format
*LSU*: large subunit ribosomal RNA
MCMC: Monte Carlo Markov chain
RFLP: restriction fragment length polymorphism
*SSU*: small subunit ribosomal RNA
