# Supplementary material for: Use of a bioinformatic-assisted primer design strategy to establish a new nested PCR-based method for Cryptosporidium
Source: Parasit Vectors. 2017 Oct 23;10:509. doi: 10.1186/s13071-017-2462-4 (PMC5654123; doi:10.1186/s13071-017-2462-4)
Supplement: Supplementary file 2 — Alignment of sequences of the variable D8 domain of the large subunit of nuclear ribosomal RNA gene (LSU) representing Cryptosporidium and closely related apicomplexans, alveolates and dinoflagellates. Oligonucleotide primers (LSU2040F, LSU3020R; LSU2065F and LSU2557R) designed specifically to regions flanking the variable D8 domain are indicated in green. Nucleotide differences from the majority consensus of the alignment are highlighted. Figure S2. Alignment of sequences of the variable D8 domain of the large subunit of nuclear ribosomal RNA gene (LSU) representing Cryptosporidium derived from 45 faecal DNA samples. Nucleotide differences from the majority consensus of the alignment are highlighted. Colpodella angusta was included as an outgroup. (PDF 222 kb) [file 13071_2017_2462_MOESM2_ESM.pdf]

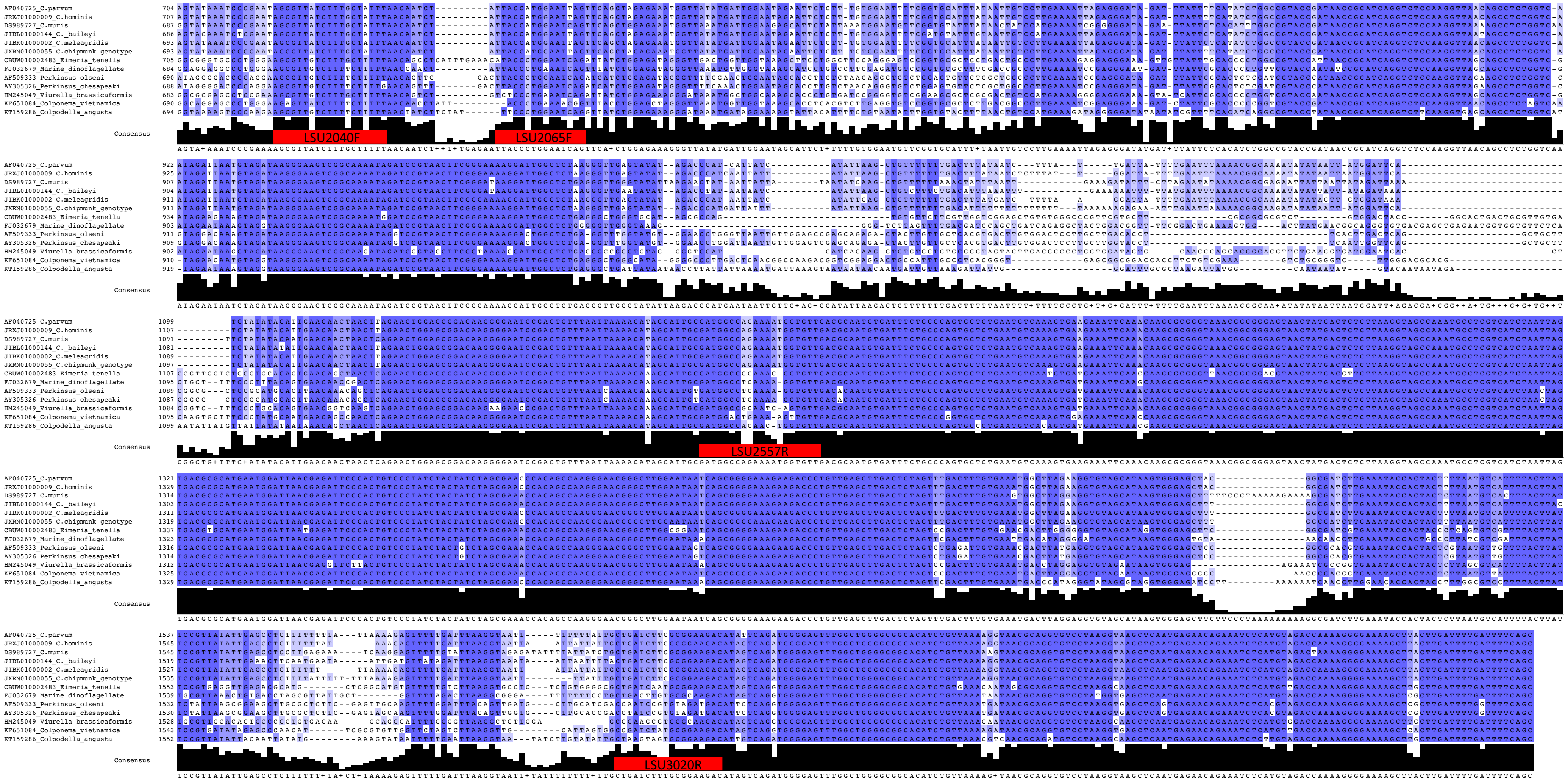
AF040725_C.parvum
JRXJ01000009_C.hominis
DS989727_C.muris
JIBL01000144_C._baileyi
JIBK01000002_C.meleagridis
JXRN01000055_C.chipmunk_genotype
CBUW01002483_Eimeria_tenella
FJ032679_Marine_dinoflagellate
AF509333_Perkinsus_olseni
AY305326_Perkinsus_chesapeaki
HM245049_Viurella_brassicaformis
KF651084_Colponema_vietnamica
KT159286_Colpodella_angusta
Consensus
LSU2040F
LSU2065F
LSU2557R
LSU3020R

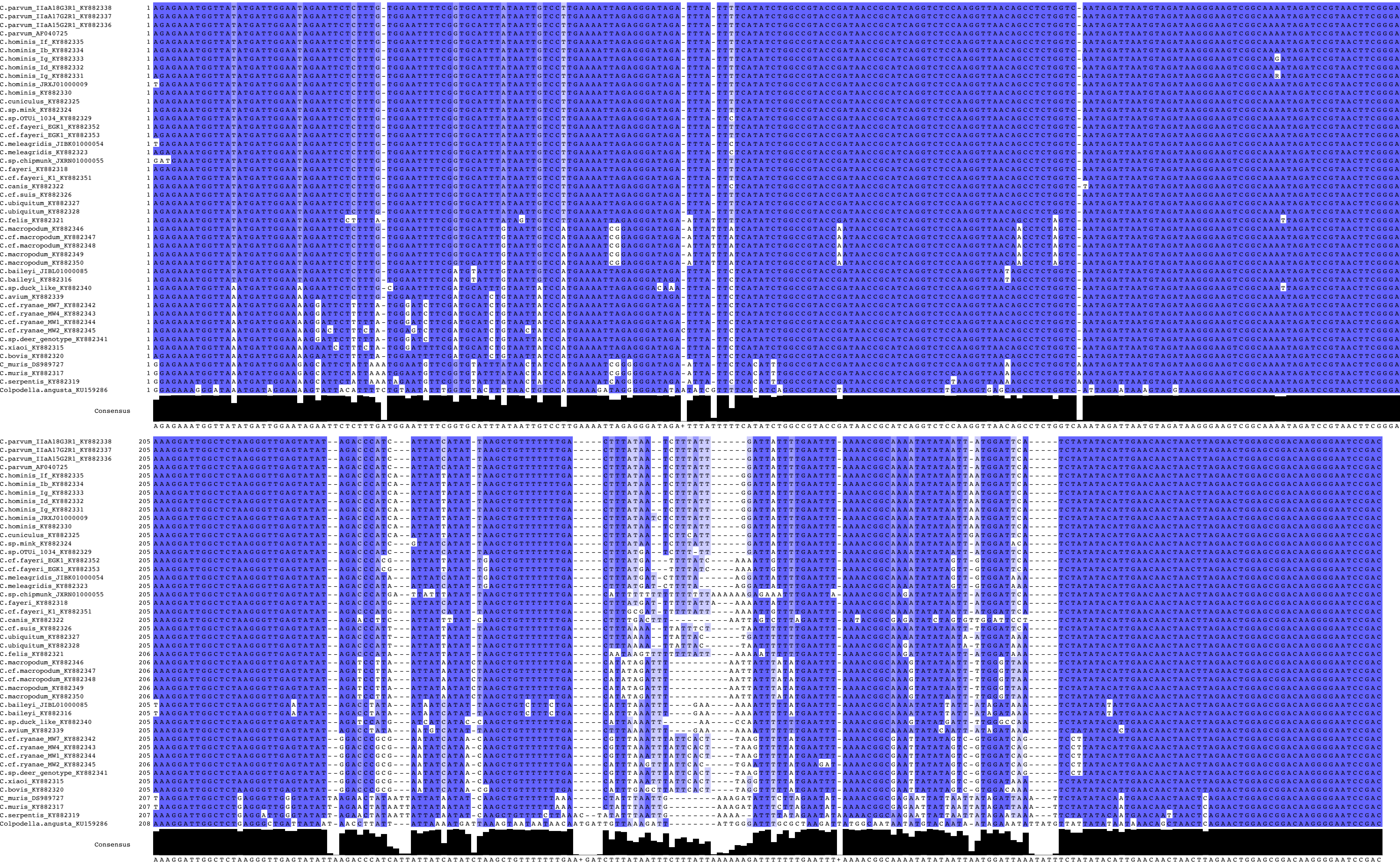
C.parvum_IIaA18G3R1_KY882338
C.parvum_IIaA17G2R1_KY882337
C.parvum_IIaA15G2R1_KY882336
C.parvum_AF040725
C.hominis_If_KY882335
C.hominis_Ib_KY882334
C.hominis_Ig_KY882333
C.hominis_Id_KY882332
C.hominis_Ig_KY882331
C.hominis_JRXJ01000009
C.hominis_KY882330
C.cuniculus_KY882325
C.sp.mink_KY882324
C.sp.OTUi_1034_KY882329
C.cf.fayeri_EGK1_KY882352
C.cf.fayeri_EGK1_KY882353
C.meleagridis_JIBK01000054
C.meleagridis_KY882323
C.sp.chipmunk_JXRN01000055
C.fayeri_KY882318
C.cf.fayeri_K1_KY882351
C.canis_KY882322
C.cf.suis_KY882326
C.ubiquitum_KY882327
C.ubiquitum_KY882328
C.felis_KY882321
C.macropodum_KY882346
C.cf.macropodum_KY882347
C.cf.macropodum_KY882348
C.macropodum_KY882349
C.macropodum_KY882350
C.baileyi_JIBL01000085
C.baileyi_KY882316
C.sp.duck_like_KY882340
C.avium_KY882339
C.cf.ryanae_MW7_KY882342
C.cf.ryanae_MW4_KY882343
C.cf.ryanae_MW1_KY882344
C.cf.ryanae_MW2_KY882345
C.sp.deer_genotype_KY882341
C.xiaoi_KY882315
C.bovis_KY882320
C_muris_DS989727
C.muris_KY882317
C.serpentis_KY882319
Colpodella.angusta_KU159286
Consensus
1
205
206
207
208
